# AMPK Signaling Involvement for the Repression of the IL-1β-Induced Group IIA Secretory Phospholipase A2 Expression in VSMCs

**DOI:** 10.1371/journal.pone.0132498

**Published:** 2015-07-10

**Authors:** Khadija El Hadri, Chantal Denoyelle, Lucas Ravaux, Benoit Viollet, Marc Foretz, Bertrand Friguet, Mustapha Rouis, Michel Raymondjean

**Affiliations:** 1 Sorbonne Universités, Université Pierre et Marie Curie, Biological Adaptation and Ageing (B2A) CNRS UMR8256/INSERM ERL-U1064, F-75005 Paris, France; 2 Université Paris Diderot, Sorbonne Paris Cité, BFA CNRS UMR8251, 4 Rue MA Lagroua Weill Hallé, 75013 Paris, France; 3 Institut Cochin, Inserm U1016, Paris, France; 4 CNRS, UMR 8104, Paris, France; 5 Université Paris Descartes, Sorbonne Paris Cité, Paris, France; Georgia State University, UNITED STATES

## Abstract

Secretory Phospholipase A2 of type IIA (sPLA2 IIA) plays a crucial role in the production of lipid mediators by amplifying the neointimal inflammatory context of the vascular smooth muscle cells (VSMCs), especially during atherogenesis. Phenformin, a biguanide family member, by its anti-inflammatory properties presents potential for promoting beneficial effects upon vascular cells, however its impact upon the IL-1β-induced sPLA2 gene expression has not been deeply investigated so far. The present study was designed to determine the relationship between phenformin coupling AMP-activated protein kinase (AMPK) function and the molecular mechanism by which the sPLA2 IIA expression was modulated in VSMCs. Here we find that 5-aminoimidazole-4-carboxamide-1-β-D-ribonucleotide (AICAR) treatment strongly repressed IL-1β-induced sPLA2 expression at least at the transcriptional level. Our study reveals that phenformin elicited a dose-dependent inhibition of the sPLA2 IIA expression and transient overexpression experiments of constitutively active AMPK demonstrate clearly that AMPK signaling is involved in the transcriptional inhibition of sPLA2-IIA gene expression. Furthermore, although the expression of the transcriptional repressor B-cell lymphoma-6 protein (BCL-6) was markedly enhanced by phenformin and AICAR, the repression of sPLA2 gene occurs through a mechanism independent of BCL-6 DNA binding site. In addition we show that activation of AMPK limits IL-1β-induced NF-κB pathway activation. Our results indicate that BCL-6, once activated by AMPK, functions as a competitor of the IL-1β induced NF-κB transcription complex. Our findings provide insights on a new anti-inflammatory pathway linking phenformin, AMPK and molecular control of sPLA2 IIA gene expression in VSMCs.

## Introduction

Phenotypic modulation of vascular smooth muscle cells (VSMCs) observed in the pathogenesis of vascular diseases such as hypertension, restenosis, aneurysm and atherosclerosis is characterized by the downregulation of the expression of VSMC-specific marker genes concomitant with the upregulation of the expression of genes regulating proliferation, migration and secretion of inflammatory mediators [1,2,3]. Atherosclerosis is a complex pathological process and accumulating evidences indicate that cardiovascular disorders are closely associated with chronic inflammation linked most often to metabolic syndrome which includes abdominal obesity, atherogenic dyslipidemia, elevated blood pressure and insulin resistance [4,5]. *In vivo* correlative data indicate that VSMCs, beside macrophages, play also an important role in the initiation of atherosclerosis [6]. After migration from the media to the intima, VSMCs present atherogenic properties. Hence, they are the major producers of extracellular matrix that in turn provoke cell adhesion and oxidized low density lipoproteins accumulation [7]. The initiation of atherosclerosis results from complex interactions of circulating inflammatory mediators. VSMCs, endothelial cells, and macrophages produce a myriad of proatheogenic proinflammatory and prothrombogenic molecules including lipid mediators such as various forms of eicosanoides, mainly prostaglandin E2 (PGE2) which in turn amplify the effects of cytokines [8,9]. Several observations argue for a major implication of type IIA secretory PLA2 (sPLA2-IIA) [10]. Phospholipase A2 enzymes (EC 3.1.1.4) hydrolyze ester bonds at the *sn-2* position of glyceroacylphospholipids to produce lysophospholipids and nonesterified fatty acids such as arachidonic acid (C20:4 n-6), the precursor of these proinflammatory mediators encompassing PGE2 [11,12]. The isoform sPLA2 IIA gene (*sPla2g2a*) is largely expressed in VSMCs in response to cytokines, interleukin-1β (IL-1β), IL-6 and tumor necrosis factor alpha (TNF–α) [13]. We have shown that oxysterols, PGE2, high mobility group B1 (HMGB1) and serum amyloid A (SAA) also induce sPLA2 IIA gene transcription in VSMCs [14, 9,15–16]. The interplay of various transcription factors (NF-κB, C/EBPβ, Ets, LXR, AP1) that stimulate the rat sPLA2 promoter activity by these inducers has been demonstrated [14,17]. More recently, we demonstrated that peroxisome proliferator-activated receptor (PPAR)-α,-γ,-β significantly inhibited cytokine-stimulated sPLA2 expression and secretion in VSMCs [18]. However, unlike to PPARα and γ, the inhibitory effect of PPARβ ligands was not affected by PPRE mutation. In concordance with primary results of Lee *et al* [19], we demonstrated that PPARβ ligand promotes B cell lymphoma 6 (BCL-6) binding to its specific sequence element in proximal position of the sPLA2IIA gene promoter. Repression mechanisms triggered by BCL-6 have been investigated in multiple cell systems. Interaction with histone-deacetylases, association with various corepressors SMRT/NCoR, SIN3A [20] and proximate binding to NF-κB response elements have been demonstrated [21]. Importantly, promoter structure as well condensation of binding sites driven induction of this *sPla2g2a* gene illustrate the pathological consequences of losing BCL-6 repression in time course of chronic inflammation.

Interestingly, anti-hyperglycemic agents currently prescribed to treat type 2 diabetes, i.e. biguanides such as metformin and phenformin, induce gene expression changes in the liver including *Bcl6* [22] and activate the highly conserved AMP-activated protein kinase (AMPK) [23–24]. AMPK traditionally activated by increase in cellular AMP/ATP ratio, has been proposed to function as a metabolic master switch to coordinate cellular adaptation to nutritional and environmental variations[25–26]. AMPK, considered as an energy-sensing system, occurs as heterotrimeric complexes which is only active after phosphorylation of a critical threonine (Thr 172) in the activation loop of the α catalytic subunit. Upstream kinases have been identified as the tumor suppressor liver kinase B1 (LKB1) associated to STRAD and MO25 accessory subunits [27–29] and the calmodulin-dependent protein kinase kinase-β (CaMKKβ) [30–31]. The binding of AMP to the γ subunit of AMPK hinder the dephosphorylation of Thr 172 by protein phosphatase. Recently, AMPK pathway appeared to be a critical downstream mediator for vascular adiponectin signalling in endothelial cells and macrophages [32–33]. In addition, the AMPK activator, AICAR (5-aminoimidazole-4-carboxamide-1-β-D-riboside), suppressed serum-induced proliferation of human aortic SMCs [34] and mediated the modulation of leucocyte adhesion observed onto aortic endothelial cells. Therefore, AMPK activation makes up a potential anti-atherogenic mechanism [35].

The study was performed to determine the impact of the AMPK signaling pathway in the regulation by biguanides of the sPLA2 IIA gene expression in VSMCs. As shown in the liver with phenformin, we hypothesize that BCL-6 may serve as a target for the control of the sPLA2 gene expression. We demonstrate here that AMPK activation significantly inhibited cytokine-stimulated sPLA2 promoter. Thus, the anti-inflammatory effect of AMPK could represent a promising therapeutic target to limit the sPLA2-dependent production of proinflammatory lipid mediators, namely PGE2, in the vascular wall.

## Materials and Methods

### Isolation and culture of VSMCs from rat aortas

VSMCs were isolated from thoracic aortas of adult male Wistar rats by enzymatic digestion as described previously [36,17]. Cells were seeded on dishes coated with type I collagen from calf skin (Sigma) and cultured in DMEM, 4mM glutamine, 100U/ml penicillin and 100μg/ml streptomycin, supplemented with 10% (vol/vol) fetal calf serum (Gibco BRL). Experiments were performed with confluent cells made quiescent by incubating them for 24h in serum-free medium containing 0.2% SVF before treatment with appropriate agents for 24h. The culture medium was then removed for measurements of sPLA2 activity and cells were lysed for total RNA preparation and western blotting. Experiments with animals and cell culture were conducted in accordance with guidelines of the "Haut Conseil des Biotechnologies" committee and according to the accepted project "OGM" with the agreement from October 11–2011. Agreement number: 5876. All procedures involving animal handling and their care were in accordance with the University Pierre and Marie Curie Guidelines for Husbandry of Laboratory Mice.

### PLA2 activity

sPLA2 activity was measured using the fluorescent substrate 1-hexadecanoyl-2-(1-pyrenyldecanoyl)-sn-glycero-3-phosphoglycerol (Interchim, France) as described previously [36] Total hydrolysis of the substrate obtained with 0.1 unit of PLA2 from bee venom (Sigma) was used as reference to calculate the sPLA2 activity in the samples. The basal level fluctuates following the preparation of VSMCs albeit the response to cytokine was of the same order until the 5th passage where the induction becomes less intense reflecting a progressive loss of differentiation.

### RNA extraction and qRT-PCR analysis

Total RNA was extracted by using RNAeasy kit (Qiagen) according to the supplier’s instructions. Total RNA (1μg) was used as template to synthesize for 1h at 37°C the first strand cDNA with 200 U of mouse mammary lentivirus-reverse transcriptase (RT) (Invitrogen), 100μM random primers and the buffer supplied by the manufacturer in a total volume of 20μl. The reaction was terminated by heating to 95°C for 5 min. Quantitative PCR was performed using the LightCycler LC480 (Roche Diagnostics). The PCR mix included 5 μl of each complementary DNA (cDNA) (diluted 1:25) and 300 nM of each primer in 1× LightCycler DNA SYBR Green 1 Master Mix. The forward and reverse primer sequences for cDNA were designed with the Primer Express software according to European Molecular Biology Laboratory accession numbers: the rat sPLA2-IIA, 5’-ATGGCCTTTGGCTCAAT-3’ (Ex1-2) and 5’-GCAACCGTAGAAGCCATA-3’ (Ex2); the rat cyclophilin A, 5’-TGCTGGACCAAACACAAATG-3’ (Ex4) and 5’-CTTCCCAAAGACCACATGCT-3’ (Ex5). The q-PCRs were performed using the following thermal settings: denaturation and enzyme activation at 95°C for 5 minutes, followed by 40 cycles of 95°C (10s), 60°C (15s), and 72°C (15s). Post-amplification dissociation curves were performed to verify the presence of a single amplification product and the absence of primer dimers. Controls and water blanks were included in each run; they were negative in all cases. Real-time quantitative PCR data represent the amount of each target messenger RNA (mRNA) relative to the amount of cyclophilin gene mRNA, estimated in the logarithmic phase of the PCR. Serial dilutions were used to determine the fit coefficients of the relative standard curve.

### Western blotting

VSMCs were harvested, homogenized in a lysis buffer at 4°C and centrifuged as described [18]. Proteins (20 μg/lane), evaluated using Quick Start Bradford protein assay from Biorad, were separated on SDS-PAGE gel electrophoresis (8% gel). After electroblotting onto nitrocellulose Amersham Hybond-ECL (GE Heathcare) we determined the efficiency of the protein transfer with Ponceau S (Sigma-Aldrich). After blocking with 5% non-fat dry milk in Tris Buffered Saline (TBS) the membrane was incubated overnight with specific antibodies, P-AMPKα (Thr172), AMPKα, P-acetyl-CoA carboxylase (Ser79) and acetyl-CoA carboxylase, BCL-6, phospho-IκBα, β-actin, phospho-IκBα, I-κBα, phospho-p65 and p-65 NF-κB (Cell Signaling Technology) in TBS containing 0.1% tween 20. After three successive 10 min washes the blot was incubated 1 h with HRP conjugated (Sigma) secondary antibody (1:2000) in TBS containing 0.1% tween 20. Detection of immune complexes was visualized using enhanced chemiluminescence reagents onto an Image captur Las 3000 (Fujifilm).

### Plasmid transfection

VSMCs were seeded 48 h before transfection in 24 well plates at a concentration of 2x10^4^ cells per plate in DMEM (2% glutamine, 1% penicillin-streptomycin, 10% SVF). At 70% confluence cells were washed 20 min before transfection. The transfection mixture was as follow: 400ng of luciferase reporter DNA [-1153; +46]sPLA2-Luc or multimeric-NF-κB[(Ig-κB]-Luc already described, 100ng of CMVβ-galactosidase, 1.6μl of Lipofectamine plus and Lipofectamine (Invitrogen) in 200μl of OPTIMEM (Sigma) and for transactivation studies 10ng of pCMV-AMPKα2 catalytic and negative dominant expression vector were added to the mix. The cells were refed 3h later with 200μl of DMEM containing 5% SVF for 1h and then the culture medium was replaced by DMEM containing 0.2% SVF. Twenty hours later after the start of the transfection, the medium was changed and the cells were pretreated with indicated concentration of AICAR (5-aminoimidazole-4-carboxamide-1-β–D-riboside), phenformin (phenylbiguanide) for 4h, then stimulated with IL-1β (10ng/ml) and the incubation was continued for a further 24h. Luciferase activity was measured using luciferase reporter assay kit (PROMEGA), with signal detection for 12s by a luminometer (Berthold, Pforzheim, Germany) and normalized by dividing the relative light units by β-galactosidase activity. The degree of induction was calculated relative to the control (minus IL-1β).

### Statistical analysis

All quantitative data are presented as mean of at least 3 independent experiments ± SEM. Statistical analyses were performed using an unpaired Student’s t-test and comparisons involving repeated measurements were analyzed by repeated-measures ANOVA followed by Bonferroni posttest. Results are expressed as the means +/- SE. Differences were considered significant at *P<0*.*05*.

## Results

### AICAR and phenformin activate AMPK in rat VSMCs

**Fig 1:**
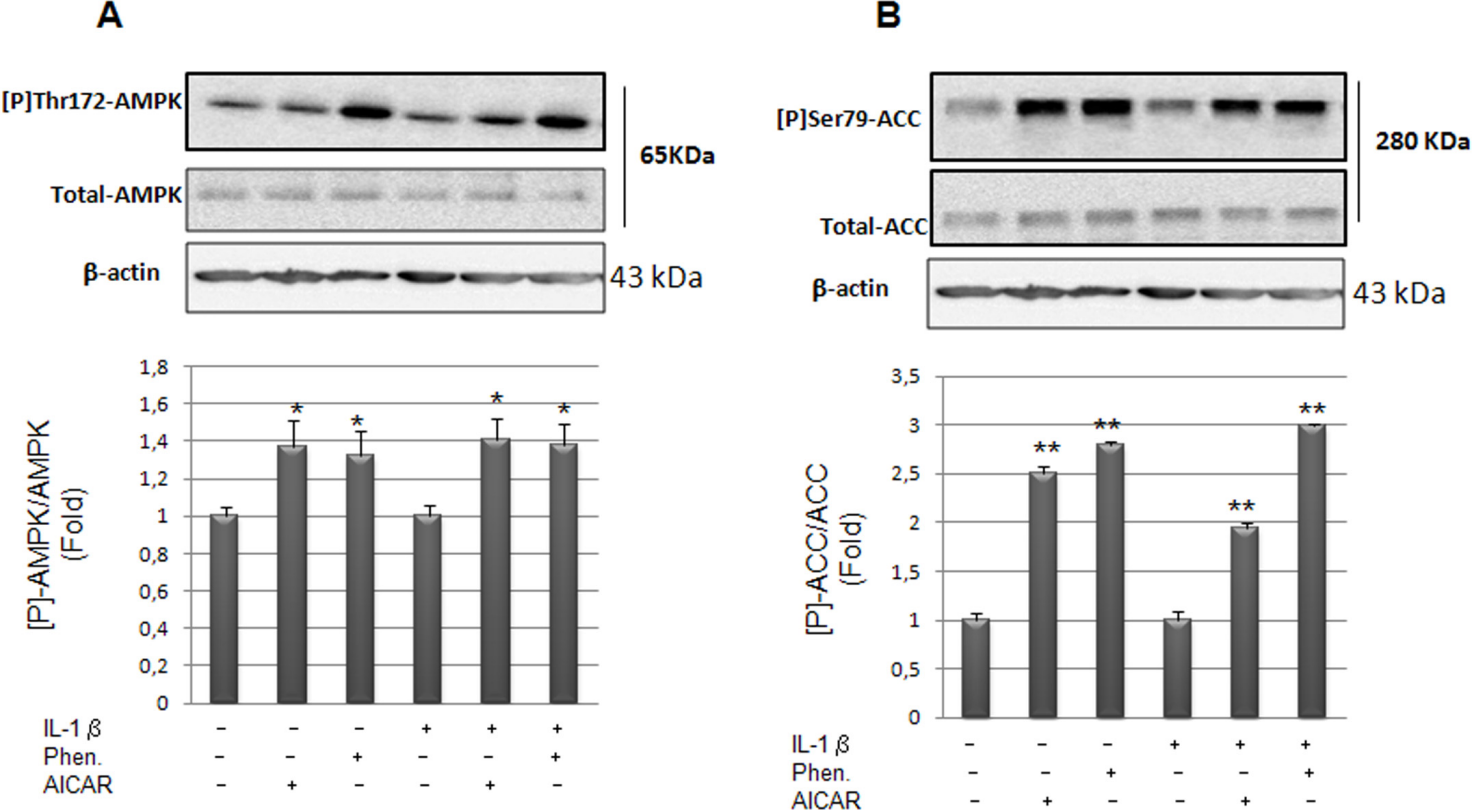
AICAR and phenformin treatments induce the phosphorylation of AMPK and ACC in isolated rat VSMCs in primary culture. After 1 hour pretreatment with AICAR (2mM) or phenformin (1mM), the cells were incubated or not with IL-1β (10ng/ml) for 30 minutes. Total proteins (20μg/lane) were separated by SDS PAGE and western blotted with specific antibodies (part A) against phospho-AMPK, total AMPK (65 KDa), and (part B) phospho-ACC, total ACC. β–actin was used as loading control. Representative blots of 3 independent experiments are shown. Data of the quantification are expressed as mean +/- SEM. *, *p* < 0,05; **, *p* < 0,01, AICAR or Phenformin treated *vs* control cells.

Experiments were conducted with primary cultures of VSMCs isolated from rat aorta. These cells undergo phenotypic changes in response to proinflammatory conditions. Indeed, in response to proinflammatory cytokines, these cells express several biomarkers of inflammation such as VCAM-1, MCP1, extracellular metalloproteinases and acute phase enzymes such as secreted sPLA2 and COX-2 [7,37–39]. Moreover, we have previously observed that prostaglandins E2 combined with IL-1β progressively synergizes the secretion of sPLA2 and causes a complete disorganization of the cytoskeletal framework [9]. To explore the effect of AMPK activation on IL-1β-induced sPLA2IIA gene expression and activity, cultured VSMCs were pretreated or not with the AMPK activators AICAR or phenformin prior IL-1β treatment. We chose to use phenformin which is more lipophilic than metformin and more efficiently internalized in cell culture in absence of cationic transporters. We performed immunoblotting to show whether treatment with AICAR or phenformin led to the activation of AMPK, characterized by the phosphorylation of Thr172 within the AMPKα subunit and of Ser79 in acetyl-coA carboxylase (ACC), a well-established target of AMPK [26]. As shown in Fig 1, treatment of VSMCs with 2mM AICAR or 1mM phenformin led to a significant increase in Thr172 AMPK and Ser79 ACC phosphorylation. In the presence of IL-1β, AICAR and phenformin induced phosphorylation of both Thr172 AMPK and Ser79 ACC. These results confirm that AICAR and phenformin stimulate AMPK signaling pathway in primary cultured rat VSMCs, consistent with previous studies showing AMPK activation by the biguanide metformin in BAECs or HUVECs [40].

### AICAR and phenformin inhibit IL-1β-induced sPLA2-IIA gene expression and activity

**Fig 2:**
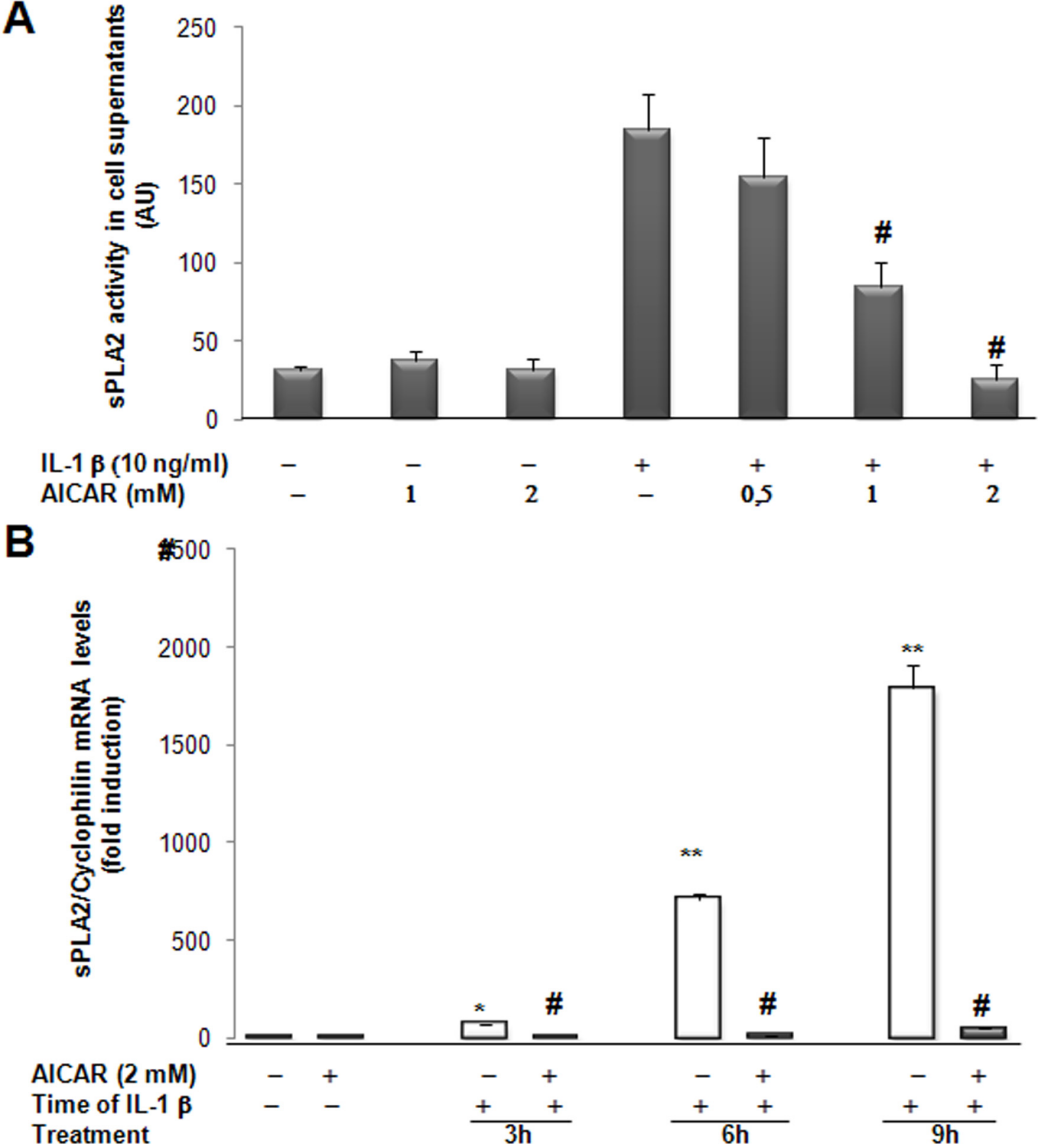
AICAR modulates IL-1β-induced sPLA2 activity and sPLA2 IIA mRNA expression in rat VSMCs. (A) VSMCs were preincubated for 4 hours with various concentrations of AICAR and then cultured in absence or presence of IL-1β (10ng/ml) for 18 hours. The sPLA2 activity was measured spectrofluorimetrically from the supernatant of the cells. (B) Cells were preincubated for 4 hours with 2mM AICAR and then treated with IL-1β for 3, 6 or 9 hours. Total RNA was extracted and real time PCR was performed to evaluate sPLA2 mRNA levels as described under Experimental procedures. Data are shown as mean +/- SEM from 3 separate experiments. *, *p*<0.01;**, *p* <0.001, IL-1β–treated *vs* control cells, IL-1β-treated *vs* control cells; #, *p*< 0.001 AICAR treated *vs* IL-1β treated VSMCs.

**Fig 3:**
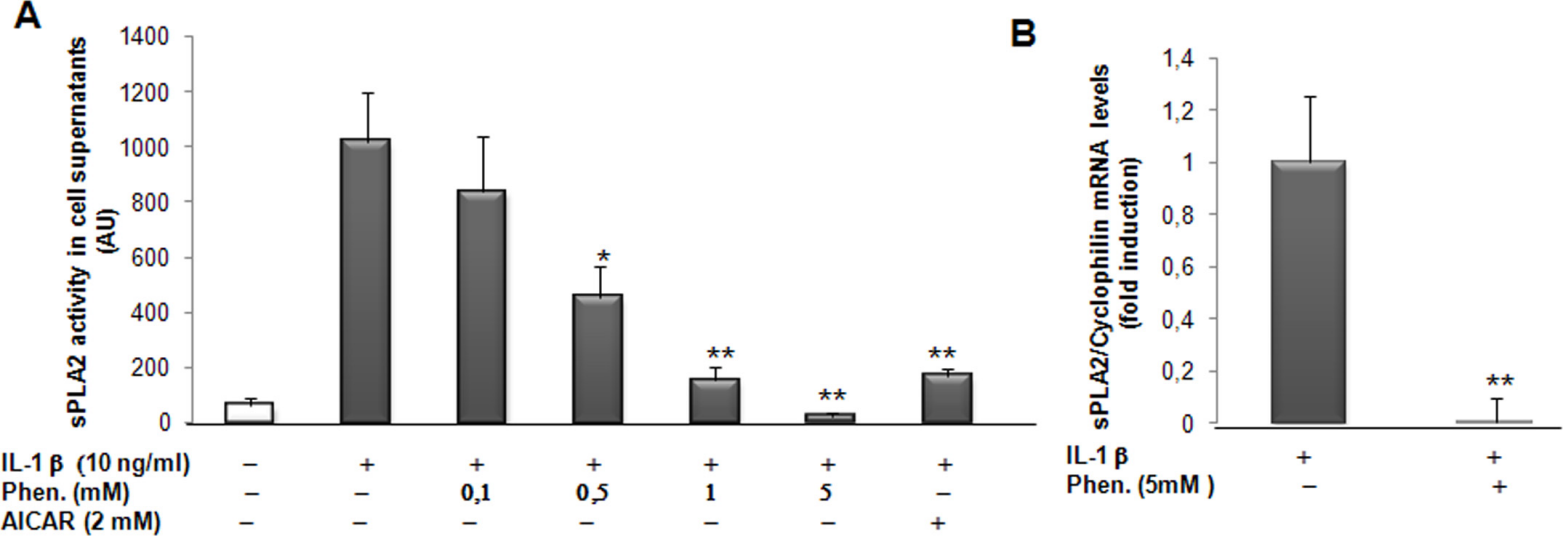
Effect of phenformin and AICAR treatment on sPLA2 activity and gene expression in rat VSMCs. VSMCs were preincubated for 4 hours with phenformin (0.1, 0.5, 1, 5mM) and AICAR (2mM) and cultured in absence or presence of IL-1β (10ng/ml) for 18 hours. (A) sPLA2 activity was measured in the supernatant of cells. (B) sPLA2 mRNA levels were determined by real time PCR analysis. Data are shown as mean +/- SEM from 3 separate experiments. *, *p*<0,01; **, *p*<0,001, IL-1β and Phenformin or AICAR treated *vs* IL-1β-treated cells.

Next, in order to investigate whether AMPK might hinder the IL-1β-induced production of sPLA2, cultured rat VSMCs were pretreated with AICAR or phenformin prior to be incubated with IL-1β. Clearly, AICAR dose-dependently caused a significant inhibition of IL-1β-induced sPLA2 activity secreted by VSMCs, whereas it has no effect on basal sPLA2 activity in the absence of IL-1β (Fig 2A). We observed that AICAR strongly inhibited IL-1β-induced sPLA2 IIA gene expression as soon as 3 hours after IL-1β treatment (Fig 2B). This result indicates that activation of AMPK pathway for 4 hours was optimal for complete inhibition of the IL-1β induced sPLA2 gene expression. Similarly, incubation with phenformin resulted in a progressive dose-dependent decrease in IL1β-induced sPLA2 activity secreted in the culture medium (Fig 3A) and significantly lowered IL-1β-induced expression of sPLA2 IIA to the basal level (Fig 3B). Our results showed that AICAR and phenformin are both potent anti-inflammatory agents that suppress sPLA2 IIA production in rat VSMCs incubated with IL-1β.

### AMPK activation represses sPLA2 IIA promoter activity in VSMCs

**Fig 4:**
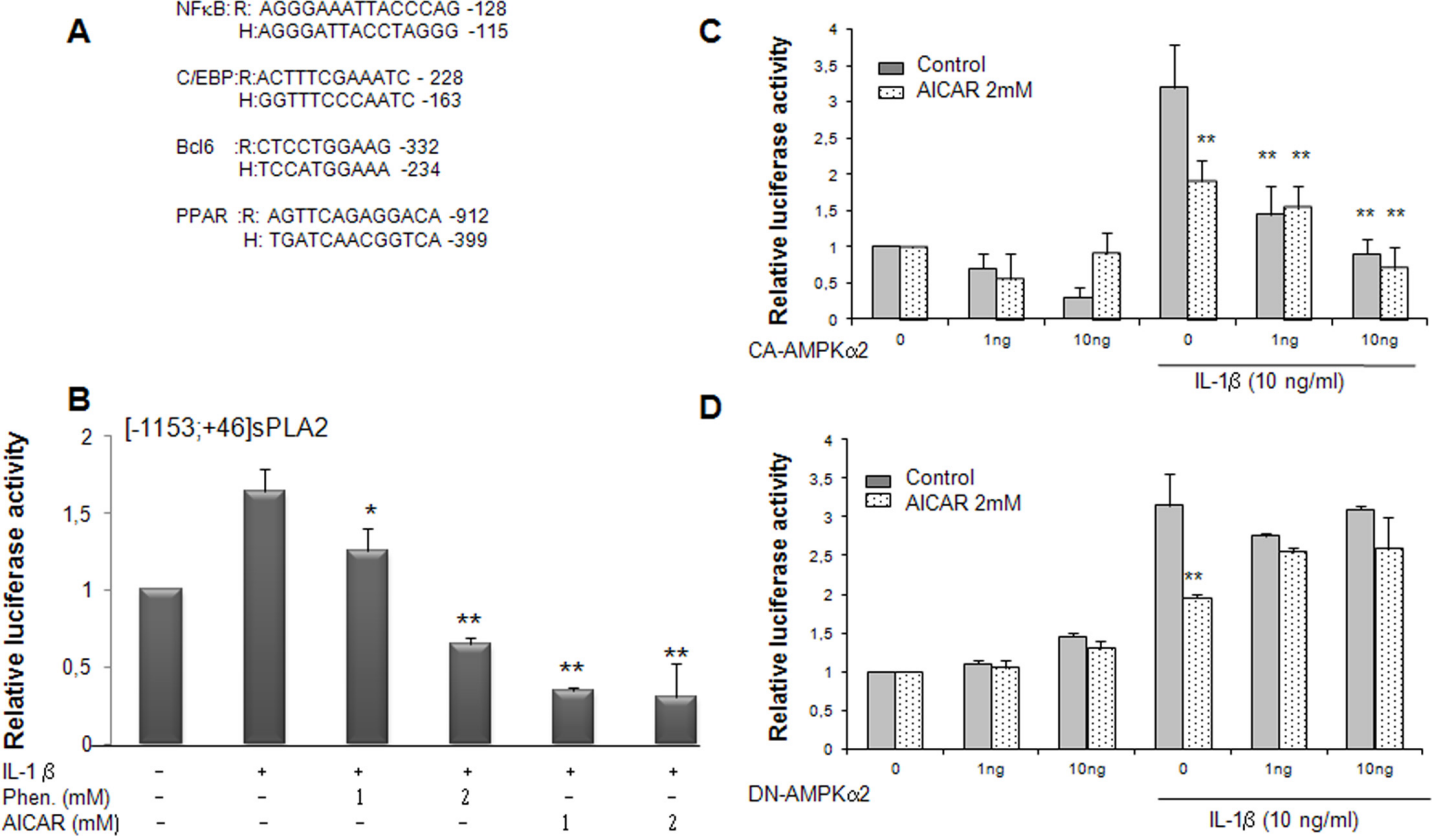
Phenformin, AICAR and overexpression of the AMPKα2 subunit inhibit the sPLA2 IIA gene promoter activity in VSMCs. (A) Alignment of rat (R) and human (H) NFκB, C/EBP, BCL-6, PPAR binding sequences of the sPLA2 IIA gene promoters. (B) VSMCs were transitory transfected with Lipofectamine plus with the sPLA2-Luc reporter plasmid: rat [-1153; +46]sPLA2 IIA-Luc construct. Cells were pretreated with phenformin (1 and 2 mM) or AICAR (1 and 2 mM) for 4 hours before prolonged incubation for 18 hours with or without IL-1β (10 μg/ml). (C and D) VSMCs were cotransfected with 1 and 10 ng of pCMV AMPKα2 constitutively active or dominant negative expression vector. As a control cells were transfected with empty pcDNA3. After transfection, cells were treated or not with IL-1β and AICAR (2mM) for 18 hours. The relative luciferase activities were calculated after normalization to β-galactosidase activity. Results (mean +/- SEM) were from 3 independent experiments. *, *P*<0.05; **, *P*<0.01 compared with IL-1β treated cells.

Since, phenformin and AICAR modulate IL1β-induced sPLA2 IIA gene expression in rat VSMCs, these results prompted us to examine the ability of AMPK to regulate the sPLA2 promoter activity. We have previously characterized the interplay of various transcription factors involved in the modulation of sPLA2 promoter activity [17–18]. Interestingly, all the binding sites were conserved and perfectly aligned between human and rat promoters (Fig 4A). A reporter construct encompassing the rat sPLA2 promoter ([-1153; +46]sPLA2-Luc) was transiently transfected into VSMCs preincubated with phenformin or AICAR for 4 hours prior to the treatment with IL-1β (10ng/ml) for 18 hours. We observed that the activity of the sPLA2 promoter was significantly decreased in a dose dependent manner with phenformin and drastically with AICAR (Fig 4B). In an attempt to determine the role of AMPK activation on the sPLA2 promoter activity, we expressed constitutively active (CA-AMPK) or dominant negative (DN-AMPK) AMPK mutants in VSMCs [41]. Expression of CA-AMPK strongly decreased the [-1153; +46]sPLA2 promoter induction by IL-1β (Fig 4C). Addition of 2mM AICAR in the cell culture medium did not show additive inhibitory effect with expression of CA-AMPK, indicating that AICAR signals through the AMPK signaling pathway to inhibit sPLA2 promoter activity. In contrast, expression of DN-AMPKα2 has no effect on basal and IL-1β-induced sPLA2 promoter activity but severely blunted the inhibitory action of AICAR on IL-1β-induced sPLA2 promoter activity, excluding off-target effect of AICAR (Fig 4D). Altogether, the experiments indicate that activation of AMPK exerts a repressive effect on IL-1β-stimulated expression of the type IIA sPLA2 gene in VSMCs. As a consequence, AMPK stimulation may reduce the extended production of proinflammatory mediators as lysophospholipids and arachidonic acid.

### Role of BCL6 as repressor stimulated by AICAR

**Fig 5:**
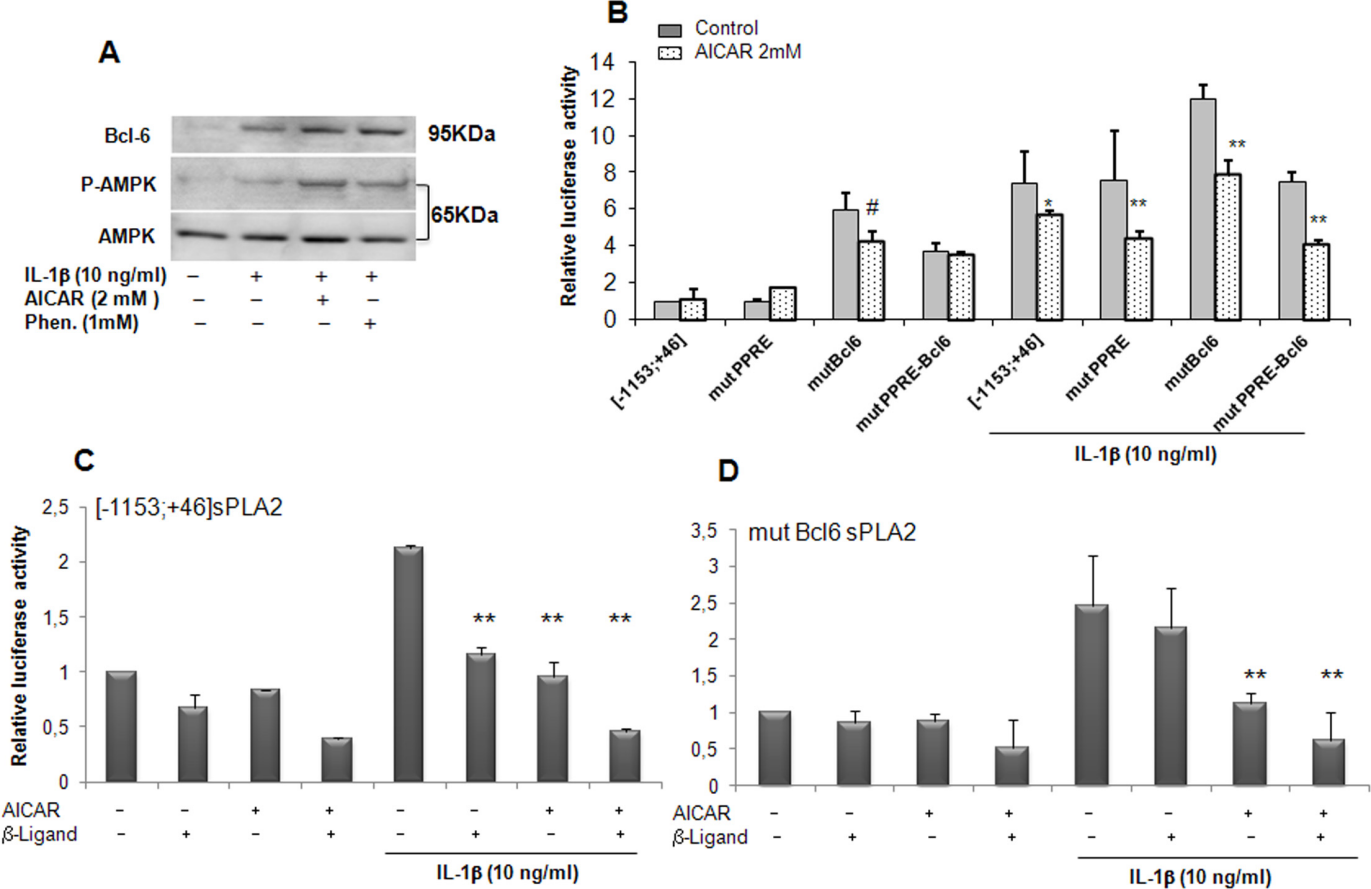
sPLA2 gene promoter inhibition is independent of the BCL-6 binding site in VSMCs. (A) protein expression was assessed by Western blot analysis, BCL-6 (95 KDa), phospho-AMPK-Thr ^172^ and AMPK (65 KDa). VSMCs were treated with IL-1β (10ng/ml) alone or with AICAR (2mM) or phenformin (1mM) for 18 hours. (B) VSMCs were transfected with the sPLA2-Luc reporter plasmids: [-1153;+46]sPLA2Luc, mutated PPREsPLA2 (mutPPRE), mutated BCL-6 site (mutBcl6), double-mutated version of the sPLA2 promoter (mutPPRE-Bcl6) and VSMCs were then treated as in Fig 4. (C) VSMCs were transfected with the [-1153;+46]sPLA2Luc plasmid, treated with PPARβ ligand (L165041, 10mM) or 2mM AICAR or both 4 hours before addition or not of IL-1β for an additional 18 hours. (D) VSMCs were transfected with BCL-6-mutated sPLA2 version, (mBCL-6[-1153;+46])sPLA2Luc construct and incubated as in part C. Data are expressed as mean +/-SEM of 4 experiments. #, P<0.05 (compared with control) and *, *P*<0.05; **, *P*<0.01 compared with IL-1β treated.

We previously showed that VSMCs expressed the proto-oncogene BCL-6 and demonstrated its ability to repress the expression of sPLA2 IIA [18], in concordance with results reported with another proinflammatory gene, MCP1 in macrophages [19]. We first determined the expression of BCL-6 in rat VSMCs by Western blot analysis. As already observed its expression was slightly augmented in the presence of IL-1β. BCL-6 expression was further increased in the presence of AICAR and phenformin concomitantly with AMPK signalling activation (Fig 5A). This finding led us to specifically consider the repressor, BCL-6, as a potential target of the AMPK pathway. We previously demonstrated that PPARβ agonist (L165041) elicited the binding of BCL-6 to the sPLA2 promoter and led to its inhibition under inflammatory conditions [18]. In an attempt to examine the role of BCL-6 binding site in the regulation of sPLA2IIA promoter transcription, VSMCs were transiently transfected with the [-1153; +46]sPLA2 promoter in which BCL-6 binding site was mutated in combination or not with mutation in the PPRE binding site. Mutation of BCL6 binding site in the [-1153; +46]sPLA2 promoter strongly increased sPLA2IIA promoter activity in basal condition, confirming the repressive role of BCL6 on sPLA2 gene expression. Interestingly, incubation of VSMCs with AICAR yielded to the same levels of inhibition observed with mutPPRE or the mutBCL-6 constructs as the wild type construct, [-1153; +46]sPLA2 in basal and IL-1β-stimulated conditions, suggesting that BCL-6 and PPRE binding sites are not necessary for the repression of sPLA2IIA promoter by AMPK (Fig 5B). As we previously demonstrated that PPARβ agonist (L165041) induced a BCL-6 binding to the sPLA2 promoter under inflammatory conditions, we performed transient transfection in the presence of either PPARβ ligand or AICAR or both. We observed that the IL-1β-induction of the [-1153; +46]sPLA2 promoter was attenuated to similar levels by PPARβ ligand and AICAR (Fig 5C). Importantly, this inhibition was further enhanced by co-treatment with PPARβ ligand and AICAR (Fig 5C). When activity of the mutBCL-6[-1153; +46]sPLA2 promoter was examined in the presence of IL1β, the inhibitory effect of PPARβ ligand was abolished but in contrast the inhibitory effect of AICAR alone or in combination with PPARβ ligand was preserved (Fig 5D). Altogether, these results suggest that the ability of AMPK to inhibit the sPLA2IIA promoter is independent on the BCL-6 binding site.

### Inhibition of IL-1β-induced NF-κB dependent transactivation by AMPK in VSMCs

**Fig 6:**
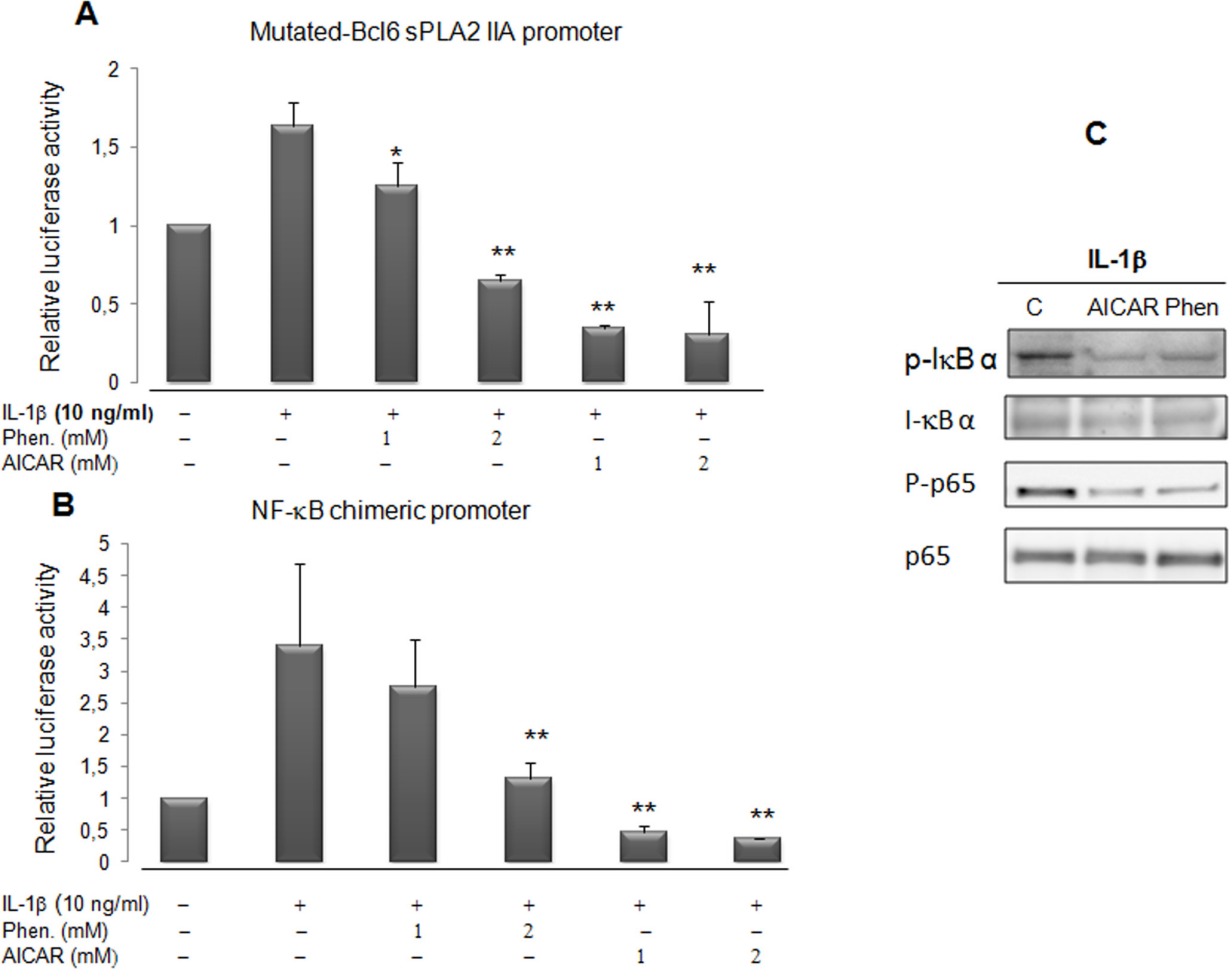
Phenformin and the activator of AMPK repress the sPLA2 gene promoter activity through NF-κB binding sites and inhibit IL-1β-induced NF-κB activation. VSMCs were transfected with either mutated-BCL-6 sPL2 IIA-Luc reporter (part A) or a NFκB-mediated-Luc-reporter (part B) construct made up a multimerized NF-κB binding site chimeric promoter and then incubated with phenformin (1 or 2 mM) and with AICAR (1 or 2 mM) in the same conditions as Fig 5. Data are expressed as mean +/-SEM of 3 experiments. *, ** *P*<0.05 and *P*<0.01 respectively compared with IL-1β treated VSMs. In part C, IkBα, phospho-IκBα, p65NF-κB and phospho-p65NF-κB expression was assessed by western blots. VSMCs were preincubated with or without AICAR (2mM) or phenformin (2mM) for 30 minutes before addition of IL-1β (10ng/ml) for 15 minutes. Then the cells were harvested and lysed as described and subjected to western blotting to detect phospho-ⅠBα, IκBα, phospho-p65NF-κB. Total. p65NF-κB is provided as a control.

The absence of AICAR and phenformin action through the BCL-6 binding site on the sPLA2 promoter (Fig 6A) prompted us to further investigate whether AMPK acts on another regulatory site. We demonstrated previously that the sPLA2 gene promoter contains a crucial NF-κB binding site at position -131bp which is crucial for the stimulation of sPLA2 promoter by IL-1β [17]. It was recently shown that AICAR and a constitutively active AMPK reduced the expression of VCAM-1 in TNFα-activated aortic endothelial cells by attenuating NF-κB acetylation [41–42]. In order to assess the impact of phenformin and AICAR on the activity of the transcriptional factor NF-κB in VSMCs, we transiently transfected VSMCs with a chimeric construct, [(IgKB)3-cona]-Luc, where 3 IgK enhancer κB sites were fused to the conalbumin promoter [43,17]. The activity of the chimeric NF-κB-Luc promoter was also strongly diminished with AICAR and phenformin treatment (Fig 6B). This result reveals that the treatment of VSMCs by either AICAR or phenformin abolished the IL-1β-induced activity of a promoter which transcription was strickly dependent upon the NF-κB pathway. To further confirm the impact of AICAR and phenformin on the activation of NF-κB transcription factor by IL-1β, we analyze degradation of NF-κB inhibitor IκBα and NF-κB translocation (Fig 6C). The blot probed with antiphospho-IκB antibody shows a phosphorylated IκBα specific band detected in IL-1β treated cells. When VSMCs where pretreated with AICAR and phenformin the impact of IL-1β upon the phosphorylation of IκBα was diminished and the IL-1β induced phosphorylation of p65 protein was clearly attenuated. These results demonstrate that activation of AMPK pathway interfere with IL-1β activation of the NF-κB signaling cascade. In consequence, the activation of proinflammatory genes, encompassing a NF-κB binding site, like sPLA2 IIA gene was strongly hindered in vascular SMCs.

## Discussion

In the present study we investigated the anti-inflammatory properties of the AMPK activators, AICAR and the antidiabetic drug phenformin, by focusing our attention on the modulation of sPLA2 IIA gene expression by inflammatory signals in VSMCs. The above findings have warranted a revisiting of the transcriptional regulation of the IL-1β-induced sPLA2 IIA gene promoter activity. Our results highlight new integrated molecular mechanisms involving AMPK signaling pathway in the inhibition of sPLA2 IIA gene expression and provide new insights through the interplay between the protooncogene BCL-6 and NF-κB transcription factors.

Inflammation in the vascular intima has emerged as a crucial factor in the pathogenesis of atherosclerosis induced by hypercholesterolemia, shear stresses of hypertension or disordered blood flow and during restenosis after coronary intervention. Circumscribed chronic inflammatory states originate from a partial destruction of cells followed by a partial healing of the tissue. The VSMCs proliferation and progressive dedifferentiation, is a critical event of the progression of vascular neointimal lesion formation. Beside chemokines, several mediators as biologically active oxidized lipids, named eicosanoids (mainly PGE2) are involved. These lipid mediators outcome from arachidonic acid (AA) and released by induced phospholipase A2 activity generate an amplification loop of the inflammation leading to a non-controlled response. The involvement of sPLA2 IIA as a key regulator in chronic and acute inflammatory diseases is well documented [44,12,45], likewise its major contribution through different pathways is clearly demonstrated in the arterial wall and VSMCs during atherosclerosis development[46–47,39]. We have taken advantage of our expertise in the field of the transcriptional regulation of the sPLA2 IIA gene expression in VSMCs to explore the action of AMPK signaling pathway. AMPK originally thought to be a major survival factor in a variety of metabolic stresses, appeared to be a critical downstream mediator of vascular adiponectin signaling [48]. In endothelial cells, it was shown that adiponectin inhibits CRP production through its ability to modulate AMPK signaling pathways that in turn suppress NF-κB activity [49]. Furthermore, AICAR reduces fatty acid-induced NF-κB activation [33] and diminishes endothelial cell proliferation under conditions of ischemia [50]. Particularly interesting, metformin (the parent phenformin biguanide) treatment of insulin resistant type 2 diabetic patients was proved to be very effective for lessening macrovascular morbidity. Pre-treatment of HUVECs with metformin was shown to have anti-inflammatory effects by suppressing TNF-α-induced IκBα degradation through the AMPK pathway [51–52]. Metformin was first reported as an AMPK agonist in hepatocytes [23], H-2K cells [51] and skeletal muscle [52] not acting through changes in energy status. However, it was recently demonstrated that metformin-induced changes in cellular AMP/ATP ratio is a pre-requisite for the activation of AMPK [30]. Particularly interesting, it was shown that activation of AMPK by AICAR suppresses the proliferation of human aortic SMCs by inducing S-15 phosphorylation and expression of p53. Induction of CDKI p21 by AICAR suggested an AMPK-dependent G1 arrest through inactivation of E2F transcription factor [34]. In the present study, we report that the expression of IL-1β-induced sPLA2IIA mRNA in VSMCs was dose-dependently inhibited by phenformin and interestingly also by resveratrol (trans-3,4’,5-trihydroxystilbene) (data not shown), a natural compound known for its beneficial effect on cardiovascular system and activation of AMPK [53–54]. This result confirms the protective activity of resveratrol in VSMCs likely by inhibiting transcription factors such as NF-κB or AP-1 following the increase of AMPK activity [55–57]. *In fine* the repressive effect that occurs through the AMPK signaling pathway affects a large coordinated network of genes involved in the inflammatory amplification loop in VSMCs. In endothelial cells, the mechanism by which metformin activates AMPK has been reported [58]

The inter-gene network of the genes affected by phenformin includes many transcription factors and specifically the transcriptional repressor BCL-6 which targets many inflammatory genes [22]. Here, we addressed the question about the action of AMPK on the transcriptional repressor BCL-6 reported as a key regulator of sPLA2 IIa gene expression. AMPK activity could *in fine* suppress inflammation through various mechanisms involving the stabilization of repressive complexes on several inflammatory gene promoters. Recently, Gongol *et al* demonstrated, in endothelial cells, that AMPKα2 facilitated the phosphorylation of PARP-1 and its dissociation from the intron 1 of the repressor POZ/zinc-finger BCL-6 and hence led to the extinction of inflammatory genes VCAM-1 and MCP-1 [59]

Nevertheless, to the best of our knowledge this is the first time that IL-1β-induced sPLA2 IIA activity is shown to be inhibited by AMPK activation mainly at a transcriptional level. Since AMPK regulates the inhibition of a network of proinflammatory genes, we focused on the molecular candidate repressor BCL-6 [60]. We previously demonstrated that sPLA2 IIA gene is a direct target of BCL-6 acting through a specific binding site in the proximal promoter. In accordance with the results reported by Lee *et al* [19] on another proinflammatory gene, MCP1, we demonstrated that PPARβ activation by PPAR ligands led to the translocation of the protooncogene BCL-6 and its binding to a specific site located between position -342 and -351bp relative to the transcription initiation start. BCL-6 could contribute to the prevention of atherosclerosis by recruiting, with a high degree of overlap, SMRT and NCoR-corepressor complexes to control the expression of inflammatory and atherogenic genes [61–62]. Precisely, these studies revealed that BCL-6 SMRT/NCoR subcistromes are still enriched for proximally bound NF-κB driven inflammatory and tissue remodelling genes in bone marrow of *ldlr*-/- mice. Effectively, the sPLA2 IIA promoter contains NF-κB and C/EBP binding sites highly conserved between rat and human (-131bp and 178bp respectively for rat and -138bp and -235bp for human, unpublished results) and a typical BCL-6 binding site located at -340bp. The organization of the sPLA2 IIA promoter is reminiscent of many promoters of inflammatory genes where NF-κB and BCL-6 sites are co-localized within a 200bp space [21]. We also observed in the present study that AMPK treatment stimulated BCL-6 expression and interestingly, we confirm, as already observed by RT-PCR and western blotting analysis [18], that BCL-6 is potentially an IL-1β-inducible transcriptional repressor, probably via the NF-κB pathway. It was also shown that DNA binding activity or stability of the BCL-6 protein is modulated by several mechanisms [63–64,8] and that BCL-6 mediated a powerful repression through a large set of interactions with tissue-specific repressors [65].

Interestingly, in contrast, to its action in the presence of the PPARβ ligand L165041, our experiments with either phenformin or AICAR did not reveal a direct role for BCL-6. We found that the mutation of the BCL-6 binding site on the sPLA2 promoter did not abolish the repression, although the inhibition of the promoter following PPARβ activation by a synthetic ligand was clearly dependent on BCL-6 DNA binding. A plausible mechanism could be that AMPK activation targets more preferentially the IL-1β-induced transcription factors such as NF-κB, C/EBPβ and AP1. According to this hypothesize transfection experiments revealed that a multimeric NF-κB synthetic promoter was perfectly repressed by phenformin and AICAR. In addition, we have shown that AMPK pathway also inhibits IL-1β phosphorylation of IκBα. In accordance, it was recently demonstrated that AMPK could also play a key role in endothelial cells as a pleiotropic inhibitor of immune response by acting through the nuclear binding activity of the NF-κB transcription factor [41]. Indeed metformin was shown to inhibit NF-κB activation by TNF-α and IL-1β via a possible AMPK pathway in endothelial cells and VSMCs [66–67].In the present study we show that AMPK activation by AICAR or Phenformin suppresses cytokine-induced NF-κB-dependent gene transcription and therefore modulates inflammatory responses in VSMCs and contributes to the partial resolution of the inflammation. Thus the findings may have significance for treatment of atherosclerotic vascular disease.

At overall, our two studies on the transcriptional repression mechanisms of the IL-1β-induced sPLA2 IIA promoter reveal at least two distinct pathways, one through the action of the PPARβ ligand, as probably PGI2 eicosanoids, in liberating sequestrated BCL-6 which binds to the BCL-6 consensus sequence and the second when AMPK is activated in blocking NF-B activation complex. AMPK activation that occurs after phenformin treatment in VSMCs could attenuate the recruitment of coactivators and inhibits IKK activity. Barroso *et al*. showed that the PPARβ agonist, GW501516 prevented TNF–α-induced expression of NF-κB target genes through AMPK activation by reducing the p300 and p65 interaction and by stimulating SIRT1 expression [63]. However, the existence of another non-exclusive inhibitory mechanisms mediated by BCL-6 may occur in VSMCs. It was shown that the C-terminal domain of BCL-6 physically interacts with the Rel-homology domain of NF-κB *in vitro* as with AP1 family members and that BCL-6 behaves as a mutual negative regulator of NF-κB target genes in diffuse large B-cell lymphomas [68–69]. The present study did not demonstrate a repressible effect stimulated by AMPK through a direct DNA binding of BCL-6, but may be consistent with a protein-protein interaction with NF-κB complexes. This leads us to hypothesize a plausible mechanism for the inhibition of the transcriptional activity of the sPLA2 IIA gene activity. In VSMCs, AMPK activation by phenformin could phosphorylate the DNA binding domain of BCL-6 which could hinder its binding to the sPLA2 IIA promoter located at -340 bp of the initiation site without affecting its protein-protein interaction with the NF-κB transcriptional factor located downstream at -131 bp. We postulate that, once phosphorylated, BCL-6 could stabilize a SMRT/NCoR repressor complex that blocks IL-1β-induced NF-κB activity and then potentially diminishes sPLA2 IIA gene transcription. In fact, our close examination of the BCL-6 sequence reveals a putative phosphorylation site by AMPK located between amino acids 11 and 16 in the N-terminal domain of BCL-6 which are conserved in human, rat, mouse and chicken: FTRHASDVLL. This putative sequence matches well with the consensus one: FxRxxSxxxL[69–70].

In addition, we cannot exclude the role of miRNA, such as miR-155, that in macrophages was shown to repress the expression of BCL-6 in attenuating NF-κB signalling in advanced atherosclerosis [71]. Interestingly, a cascade of mRNA targeted by miR-155 would be involved in the regulation of vascular inflammation as described with the use of polyphenolic compound as resveratrol [72].

The knowledge gained by this study about the sPLAIIA gene promoter will improve the overall understanding of how cytokine-induced genes are regulated. On account of the closed disposition of the regulatory elements, the study of the transcriptional activity of the promoter will allow to identify new signalling pathways. A novel repression mechanism of the cytokine-mediated induction of sPLA2 IIA in hepatocytes was recently deciphered [73]. The gene activity was blocked by the recruitment of corepressors SMRT and NCoR to the T3-liganded TRβ bound to a non canonic site located between -102bp and -82bp, on the proximal region of the rat sPLA2 IIA gene promoter. In fact, DNA binding interactions were precisely characterized in the same mapped region (from -101 to -77bp) by DNA footprinting and EMSA assays with VSMCs crude extracts (Antonio V. and Raymondjean M., unpublished results). This new report and our present study show evidence about a network of positive and negative mechanisms mediating the sPLA2 IIA promoter activity. The complexity and the overlapping of the transcription factors highlight the crucial role played by the sPLA2 IIA in the control of cell fate, i.e., proliferation, dedifferentiation and secretory status of VSMCs.

Interestingly, recently AMPK was shown to be the central target for the metabolic effects of resveratrol *in vivo* by increasing the NAD to NADH ratio, thus contributing indirectly to the stimulation of SIRT1 [74–75]. These evidences illustrate perfectly the central role played by the fuel-sensing kinase activated by various metabolic and stress conditions. More recent studies investigating the vascular consequences of AMPK deletion *in vivo* have shown that knockout of AMPKα2 contributes to neointima formation after vascular injury and moreover, upregulation of proinflammatory markers was observed in arteries of α1AMPK-knockout mice after ATII infusion [76–77].

In summary, our study highlights the mutual exclusive regulation mechanism plays by BCL-6 when therapeutic interventions by PPARβ ligands and antidiabetic drugs are administrated to patients Indisputably, more studies are needed to further understand how biguanide molecules acting through the AMPK signaling pathway may have various beneficial metabolic effects by reducing associated atherogenesis [78]. These results point to the sPLA2 IIA as a potential therapeutic target for numerous inflammatory diseases.
